# Ancestry, admixture, and pathogens in contemporaneous Neolithic farmers and foragers on the Island of Gotland

**DOI:** 10.1038/s42003-026-10498-0

**Published:** 2026-07-13

**Authors:** Magdalena Fraser, Federico Sanchez-Quinto, Emrah Kırdök, Kristiina Ausmees, Gülşah Merve Kılınç, Maximilian Larena, Leonardo Correa-Mendoza, Adrien Le Meur, Antonio Blanchet, Nora Bergfeldt, Eduardo Arrieta-Donato, Mariana Escobar-Rodríguez, Anders Götherström, Karla Lozano-Gonzalez, Israel Aguilar-Ordoñez, Helena Malmström, Kjel Knutsson, Paul Wallin, Nicolas Rascovan, Jan Storå, Mattias Jakobsson

**Affiliations:** 1 Human Evolution, Department of Organismal Biology, Uppsala University, Uppsala, Sweden; 2 International Laboratory for Human Genome Research, National Autonomous University of Mexico (UNAM), Queretaro, Mexico; 3 Department of Biotechnology, Faculty of Science, Mersin University, Yenişehir, Turkey; 4 Department of Information Technology, Uppsala University, Uppsala, Sweden; 5 Department of Biological Sciences, Middle East Technical University, Ankara, Turkey; 6 Center for Genomic Sciences, National Autonomous University of Mexico, Cuernavaca, Mexico; 7 Microbial Paleogenomics Unit, Institut Pasteur, Université de Paris Cité, Paris, France; 8 Centre for Palaeogenetics, Stockholm University, Stockholm, Sweden; 9 Department of Zoology, Stockholm University, Stockholm, Sweden; 10 Department of Bioinformatics and Genetics, Swedish Museum of Natural History, Stockholm, Sweden; 11 Department of Archaeology and Classical Studies, Stockholm University, Stockholm, Sweden; 12 Centre for Developmental Neurobiology, King’s College London, London, UK; 13 oriGen Project, Tecnologico de Monterrey, Monterrey, Mexico; 14 Department of Archaeology, Ancient History and Conservation, Uppsala University, Uppsala, Sweden; 15 Department of Archaeology, Ancient History and Conservation, Uppsala University-Campus Gotland, Visby, Sweden; 16 Osteoarchaeological Research Laboratory, Department of Archaeology and Classical Studies, Stockholm University, Stockholm, Sweden

**Keywords:** Population genetics, Evolutionary genetics

## Abstract

Two archaeological cultural complexes; the Neolithic Funnelbeaker culture (FBC) and the Pitted ware culture (PWC), coexisted on Gotland for over 500 years, between ~3300 and 2800 calBCE. The ancestry of the FBC farmers and PWC marine foragers largely aligns with European Neolithic Farmers and European Mesolithic foragers, respectively, but the direct interactions between the groups on Gotland is not understood. We present a Middle Neolithic (MN) high-coverage genome and a Late Neolithic (LN) low-coverage genome from the Ansarve FBC dolmen. We investigate ancestry, admixture, and pathogens among these MN farmers (*n* = 6), foragers (*n* = 19), and the LN individual. We find that recent gene-flow between farmers and foragers could have taken place, although most gene-flow happened prior to their coexistence on the island. We also find evidence of different *Yersinia pestis* strains in the three cultural groups, showing that the pestis was widespread among groups with different subsistence strategies.

## Abstract (web summary)

Neolithic farmers and foragers in Gotland coexisted for 500 years, ancient DNA suggests limited intermixing, shared ancestry with mainland groups, isolation, and evidence of distinct *Yersinia pestis* strains in both populations.

## Introduction

Due to the rapid advancement in archaeogenomics, we now know that some of the present-day patterns of genomic variation in Northern Europe were shaped by several major prehistoric demographic events^1^. These include the Palaeolithic and Mesolithic post-glacial dispersal of the pioneer hunter-gatherer groups northwards over the continent, the displacement and assimilation of Mesolithic hunter-gatherers (HGs) during the expansion of Early Neolithic farmer (ENF) groups^2^, followed by the expansion of groups of the Corded Ware complex (CWC), bringing the so-called Steppe-ancestry genetic component into the area^3,4^.

**Fig. 1:**
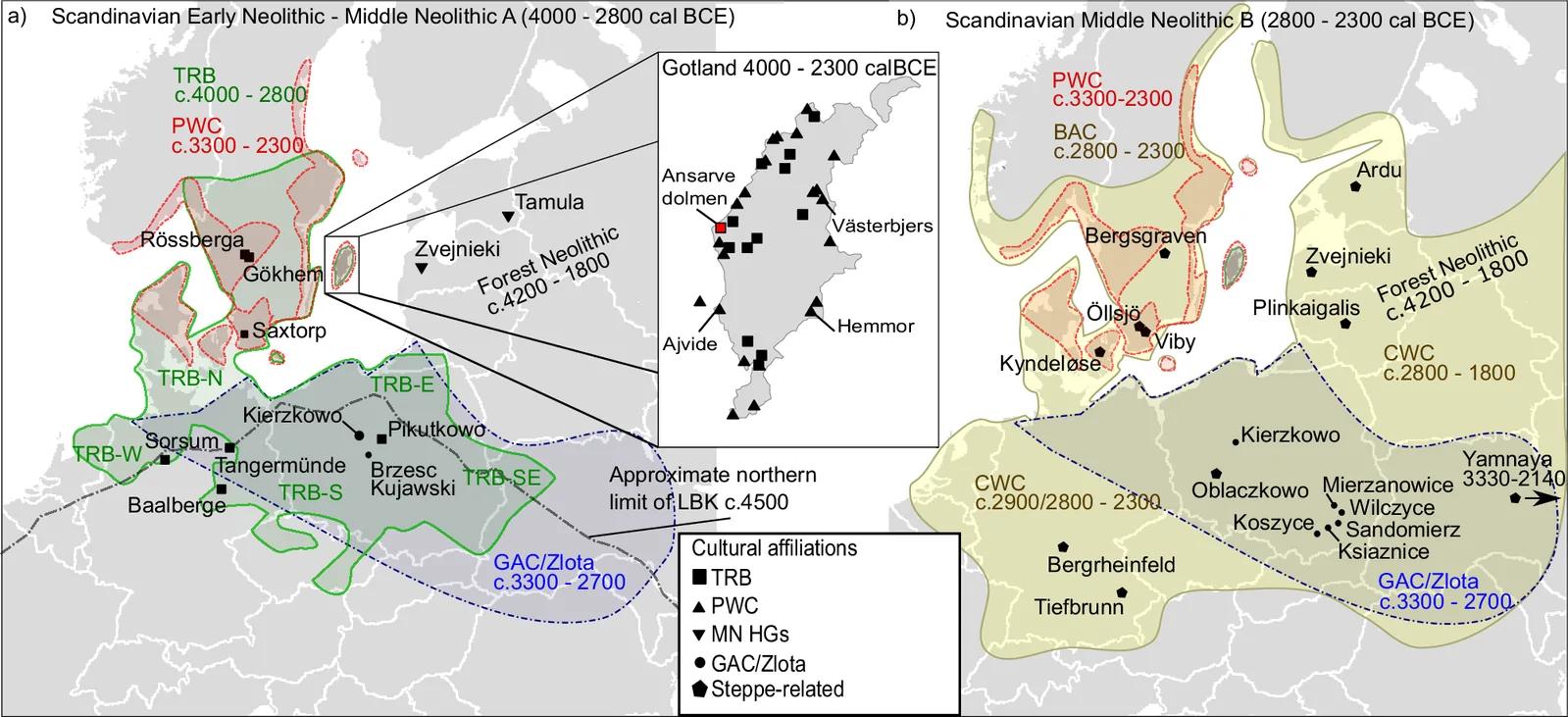
Map of Northern and Central Europe with approximate distribution and timelines for the TRB (Trichterbecher) culture, PWC (Pitted Ware) culture, Forest Neolithic cultures, GAC (Globular Amphorae)/Zlota cultures, and BAC/CWC (Battle Axe/Corded Ware) Steppe-related cultures. **a** Distribution during the Scandinavian Early Neolithic to Middle Neolithic A periods, relevant contemporaneous sites with comparative data are shown in black. The dashed gray line shows the northern limit of the LBK (Linearbandkeramik) culture. Cultural distributions are shown in green (TRB), red (PWC), and blue (GAC/Zlota cultures). Insert: Map of Gotland showing the location of TRB-sites (squares) and PWC-sites (pyramids). The Ansarve dolmen from this study is shown in red; sites with comparative data are labeled. **b** Distribution during the Scandinavian Middle Neolithic B period. The PWC and GAC/Zlota cultures show the same distribution and color as in (**a**). Distribution of BAC/CWC (Battle Axe/Corded Ware) cultures is shown in yellow, contemporaneous sites with comparative data are shown in black. Cultural timelines in both (**a**) and (**b**) are presented as calBCE, see Supplementary Data 1 for comparative data.

Although the overarching patterns of migration during the European Neolithic are known, the demographic and social development in many local regions of Europe has not been fully explored and may therefore show different and unique patterns of interactions between foragers and farmers. The island of Gotland [Fig. 1] is unique in this way; though situated in the middle of the Baltic Sea, it mirrors the general Neolithic developments seen in southern Scandinavia^2,5–9^ but also displays local developments^10–14^.

The Scandinavian peninsula was initially populated from the south by Mesolithic HG-groups displaying genetic ancestry from western HGs (WHGs)^8^, but also from the northeast by HG-groups exhibiting an eastern genetic ancestry (EHG), resulting in varying degrees of WHG and EHG-admixture into the regional Scandinavian HG-groups (SHGs), which is also seen on Gotland^14,15^. The Neolithization in southern Scandinavia (including Gotland) is associated with the Funnel Beaker/Trichterbecher cultural complex (TRB, 4000–2800 calBCE), e.g., refs. ^16–22^. The transition is associated with the appearance of groups exhibiting genetic ancestry mainly from the ENF, but also exhibiting some degrees of ENF and HG-admixture^2,5,7,8,11,23,24^. It has recently been shown that TRB-associated individuals from present-day Denmark, Sweden, and Poland form a genetic subcluster, suggesting a northeastern European origin for the source population of the Scandinavian TRB groups^8^. Around c. 3600 calBCE^25^, a new megalithic burial tradition was manifested in the TRB-complex in the form of dolmens and passage-graves^21,26–29^, indicating continued long-range contacts within the widespread TRB-sphere [Fig. 1a].

It has been suggested that the forager admixture components in the Scandinavian TRB-groups were WHG-related and that admixture mainly occurred prior to reaching Scandinavia^8^, though local admixture with SHG-related groups has also been suggested^5^. However, Neolithic period forager groups were also present in much of Scandinavia, i.e., the marine Pitted Ware culture (PWC, 3300–2300 calBCE), that exhibit contacts with local TRB-groups, making it possible that there were direct interactions between these contemporaneous groups. The PWC thrived along the coastal areas of present-day northeastern Denmark up to middle Sweden, southern Norway, on Gotland, and the western islands of the Baltic Sea [Fig. 1a]^19,30–33^. While the culture was widespread, genetic analyses have previously only been conducted for Gotlandic PWC-associated individuals, revealing mainly SHG-ancestry with a slight degree of ENF-admixture, e.g., ref. ^10^. In present-day Denmark, two individuals contemporaneous with the Gotlandic PWC have been found that display only SHG-ancestry (and no ENF-admixture)^8,34^, suggesting differences in the demographic history.

PWC-associated human remains are rare in the archeological record of mainland Scandinavia. However, the remains of both TRB- and PWC-associated individuals are represented in the archeological record on the island of Gotland. Until recently, it was suggested that Gotland in the Middle Neolithic was settled by one group with seasonal subsistence practices that adopted farming and animal husbandry^32,35^. However, archaeogenetic studies have shown that individuals from the two archeological groups—associated with different subsistence economies and cultural practices—exhibit distinct genetic variability, and thus, different demographic history and ancestry.^11,24^. Although they coexisted on Gotland for over 500 years^12^ [Supplementary Section 1], the social contacts and, thus, possible gene-flow between the TRB and PWC groups on the island have not been fully investigated.

While at least ten sites with TRB pottery and domestic faunal remains have been found on Gotland^36,37^, only one confirmed megalithic TRB burial has been located to date, the Ansarve dolmen [Fig. 1]^12,38–41^. Previous archaeogenetic analyses of individuals buried in the Ansarve dolmen revealed continuity in the male lineage over time, as well as a 2nd degree kinship relation between two males, suggesting that the burial was used by a patrilocal society^24^. These results are in accordance with the recent findings for the contemporaneous TRB on the Swedish mainland^7^ and emphasize the uniqueness of the Ansarve burial for the local TRB groups on the island.

At least twenty different PWC-settlement sites have been documented along the former Gotlandic coastline [Fig. 1], and several of them include burial grounds with many flat grave burials^32,35,42–47^. New sites are still being found^48^ which indicates that the PWC culture was abundant. Thus, the TRB and PWC complexes were contemporaneous on Gotland during the Scandinavian Middle Neolithic (MN) period between c. 3300–2800 calBCE, but after ~2700 calBCE, only the PWC complex remained.

Around c. 2800 calBCE, the archeological record changes in present-day Sweden with the appearance of the Battle Axe (BAC/CWC) cultural complex^19,44,49–53^ [Fig. 1b]. Archaeogenetic research shows that the transition coincides with the dispersal of humans with a Steppe-related ancestry component^3,6–8^. The BAC/CWC-phase overlaps with the end of the TRB phase on Gotland. Some BAC stray artifacts have been found across the island^38^, and some artifact types even in some PWC-burials^43,44,54^, although so far, no typical BAC-burials or settlements have been located. Neither has any Steppe-related admixture been detected in individuals of PWC burials, not even in those with cultural BAC influences^10^.

Interestingly, during this period of high human mobility, interaction, and cultural and social transformation in the region, pathogens, and more specifically, *Yersinia pestis*, seem to have widely spread, and several prehistoric *Y. pestis* strains have recently been reported. The earliest basal lineage to date was found in a hunter-fisher-gatherer from Riņņukalns, Latvia (3350–3100 calBCE)^55^, prior to the appearance of CWC in the Baltic area. However, repeated outbreaks of plague have been discovered in a TRB community in Falbygden and several other sites across southern Scandinavia, showing that the so-called Neolithic plague was common in these regions 5000 years ago^7,56^. Additional lineages have been discovered in two contemporaneous individuals (3340–2900 calBCE) from the Warburg Necropolis in western central Germany, of which one lineage seems to fit within the Falbygden *pestis* clade^57^. A few centuries later, and concomitant to the CWC migrations, additional strains from the so-called Late Neolithic/Bronze Age (LNBA) *pestis*-lineage arrived in the Baltic area, found in two individuals from Gyvakarai, Lithuania (2621–2472 calBCE) and Kunila, Estonia (2574–2340 calBCE), both exhibiting the Steppe-related ancestry component^58^. Thus, it has been shown that this pathogen probably played an important role during the Neolithic decline^7,56^ and its potential presence on Gotland could add new information to the demographic developments on the island during the Scandinavian Neolithic.

In this study, we reanalyze the six individuals from the Ansarve dolmen^24^, together with 19 individuals from three PWC sites on the island (Ajvide, Hemmor, and Västerbjers) presented in Coutinho et al.^10^ to elucidate the demographic development on Gotland when the TRB- and the PWC complexes coexisted. We present new high-coverage genomic data for one of the Ansarve individuals, as well as new low-coverage data for a later-dated individual buried in the dolmen. Finally, we investigate the possible presence of ancient strains of *Y. pestis* in the Gotlandic individuals.

## Results

From here on, we use the collective label “Neolithic Farmer” (NF) when referring to all archeological individuals/groups from an “Early – Middle” Neolithic context in the population genetic analyses, as genetically they broadly shared the same ENF-demographic history. For more information on labels, see Table 1, Supplementary Data 1, and Supplementary Section 4.1.

**Table 1:** Ancient individuals analyzed from Gotland showing time period, nomenclature used in this study, genome coverage, mitochondrial and Y-chromosome haplogroups, biological sex, 14C range, archeological site and parish, plus reference to data

Time period	Identifying label^a^	Sample name	Genome coverage	mtDNA haplogroup	Y-chromosome haplogroup^b^	Bio. Sex	14 C range, calBCE, 2σ^c^	Archeological site/ parish	Reference
Mesolithic	SHG_Gotland	sbj001	0.43	U4a1	I2-L68	M	**7020–6640**^**de**^	Stora Bjers, Stenkyrka	^14^
Mesolithic	SHG_Gotland	SF9	1.15	U4a2	-	F	**7410–7070**^**de**^	Stora Förvar cave, Stora Karlsö, Eksta	^14^
Mesolithic	SHG_Gotland	SF12	57.79	U4a1	-	F	**7080–6830**^**de**^	Stora Förvar cave, Stora Karlsö, Eksta	^14^
MN	SE_TRB	ans005	0.13	K1a2b	-	F	3500–3130	Ansarve dolmen, Tofta	^24^
MN	SE_TRB	ans003	0.14	T2b8	-	F	3490–3110	Ansarve dolmen, Tofta	^24^
MN	SE_TRB	ans008	1.94	J1c5	I2a1a2a1a1-S2703^f^	M	3340–3030	Ansarve dolmen, Tofta	^24^
MN	SE_TRB	ans014	2.58	J1c5	I2a1a2a1a1-S2703^f^	M	3330–2950	Ansarve dolmen, Tofta	^24^
MN	**SE_TRB**	**ans017**	**27.42**	HV0a	I2a1a2a1a1-S2703^f^	M	3330–2930	Ansarve dolmen, Tofta	**This study**
MN	SE_TRB	ans016	0.33	H7d	I2a1a2a-S2715^f^	M	2810–2580^e^	Ansarve dolmen, Tofta	^24^
MN	SE_PWC	ajv59	0.16	U5b2a2	ND	M	3010–2880^e^	Ajvide, Eksta	^5,10,14^
MN	SE_PWC	ajv36	0.97	U5b2a2	-	F	3010–2870^e^	Ajvide, Eksta	^10^
MN	SE_PWC	ajv28	1.35	U4a2	-	F	2890–2670^e^	Ajvide, Eksta	^10^
MN	SE_PWC	ajv58	2.68	U4d	I2a1a1-CTS595	M	2880–2630^e^	Ajvide, Eksta	^5,10,14^
MN	SE_PWC	ajv70	1.29	U4d	I2a1a1-CTS595	M	2870–2620^e^	Ajvide, Eksta	^5,10,14^
MN	SE_PWC	ajv54	0.91	U5b1d2	I2-M438	M	2900–2680^e^	Ajvide, Eksta	^6^
MN	SE_PWC	hem005	2.39	U5a1	-	F	3340–3020^e^	Hemmor, När	^10^
MN	SE_PWC	hem004	0.71	U4a2	I2-M438	M	3310–2910^e^	Hemmor, När	^10^
MN	SE_PWC	hem001	0.29	K1a3a	ND	M	3010–2880^e^	Hemmor, När	^10^
MN	SE_PWC	vbj012	1.63	U5b2a2	I2a1-L460^f^	M	3010–2880^e^	Västerbjers, Gothem	^10^
MN	SE_PWC	vbj006	3.89	K1a3a	I2a1-L460^f^	M	2920–2710^e^	Västerbjers, Gothem	^10^
MN	SE_PWC	vbj017	0.08	U4a2	ND	M	2920–2630^e^	Västerbjers, Gothem	^10^
MN	SE_PWC	vbj018	1.96	U5a2	I2a1-L460^f^^g^	M	2910–2690^e^	Västerbjers, Gothem	^10^
MN	SE_PWC	vbj002	0.16	U5b2a2	-	F	2880–2630^e^	Västerbjers, Gothem	^10^
MN	SE_PWC	vbj013	3.05	U4a2	I2a1-L460^f^^g^	M	2880–2630^e^	Västerbjers, Gothem	^10^
MN	SE_PWC	vbj004	4.54	U5b2a2	-	F	2880–2500^e^	Västerbjers, Gothem	^10^
MN	SE_PWC	vbj001	4.03	U4a2	-	F	2860–2470^e^	Västerbjers, Gothem	^10^
MN	SE_PWC	vbj008	0.90	U5b1d2	I2-M438	M	2860–2470^e^	Västerbjers, Gothem	^10^
MN	SE_PWC	vbj007	0.19	HV12	ND	M	2550–2300^e^	Västerbjers, Gothem	^10^
LN	**SE_LN**	**ans010**	**0.06**	U5b2a1a1	**I2a2a-PF3918**	**M**	2030–1890	Ansarve dolmen, Tofta	^12^ **and this study**

Results presented in bold text are from this study.

*MN* Middle Neolithic, *LN* Late Neolithic, *Bio* biological.

^a^Nomenclature used for the individuals analyzed in this study is based on a two-letter International Organization for Standardization (ISO) code for country, abbreviated cultural name, or the time period(s), and sample name (for individuals) or site name for groups. [For more information on labels used in this study, see Supplementary Section 4.1 and Supplementary Data 1].

^b^Y-chromosome assessment is based on ISSOG v. 15.73, July 11, 2020, Supplementary Section 3,

^c^All radiocarbon dates have been previously published using the IntCal13 atmospheric curve^118^ and Oxcal online software (v. 4.2.4 Ansarve dolmen, and v. 4.3.2 PWC)^119^. All calibrated dating results are rounded to the nearest 10th value.

^d^Mesolithic samples were recalibrated to calBCE using OxCal online v. 4.4^119^ and the intcal13 atmospheric curve^118^ (using sbj001; Ua-46147, SF12; Beta-448531, and SF9: Beta-399027) [Supplementary Data 1].

^e^Radiocarbon dates have been adjusted for the reservoir effect 70 ± 40 after Eriksson et al.^120^.

^f^The Y-chromosome haplotype has been updated in ISSOGG v. 15.73, July 11, 2020, Supplementary Table 4.

^g^The Y-chromosome haplotype has been restricted from previous published results, Supplementary Table 4.

### Genome sequencing and population structure

We generated a 27.42× coverage Uracil-DNA glycosylase (UDG) treated genome for the juvenile male SE_TRB_ans017 (3330–2930 calBCE) presented in Sánchez-Quinto et al.^24^. As well as, 0.06× genome coverage for the adult individual SE_LN_ans010 (2030–1890 calBCE) from later use of the burial presented in Fraser et al.^12,13^, which we here could determine to be male [“Methods,” Table 1 and Supplementary Tables 1 and 2]. The sequence data exhibit characteristic properties of ancient DNA: short fragment size, and cytosine deamination at the ends of fragments^59^ between 0.15 and 0.58, and contamination levels were found to be low [Supplementary Fig. 1 and Supplementary Tables 2 and 3]. The uniparental markers for SE_TRB_ans017 (HV0a and I2a1a2a1a1-S2703) and SE_LN_ans010 (U5b2a1a1) coincides with previously presented results^12,24^, we were also able to determine the Y chromosome hg for SE_LN_ans010 to be I2a2a-PF3918 (Table 1 and Supplementary Table 4).

**Fig. 2:**
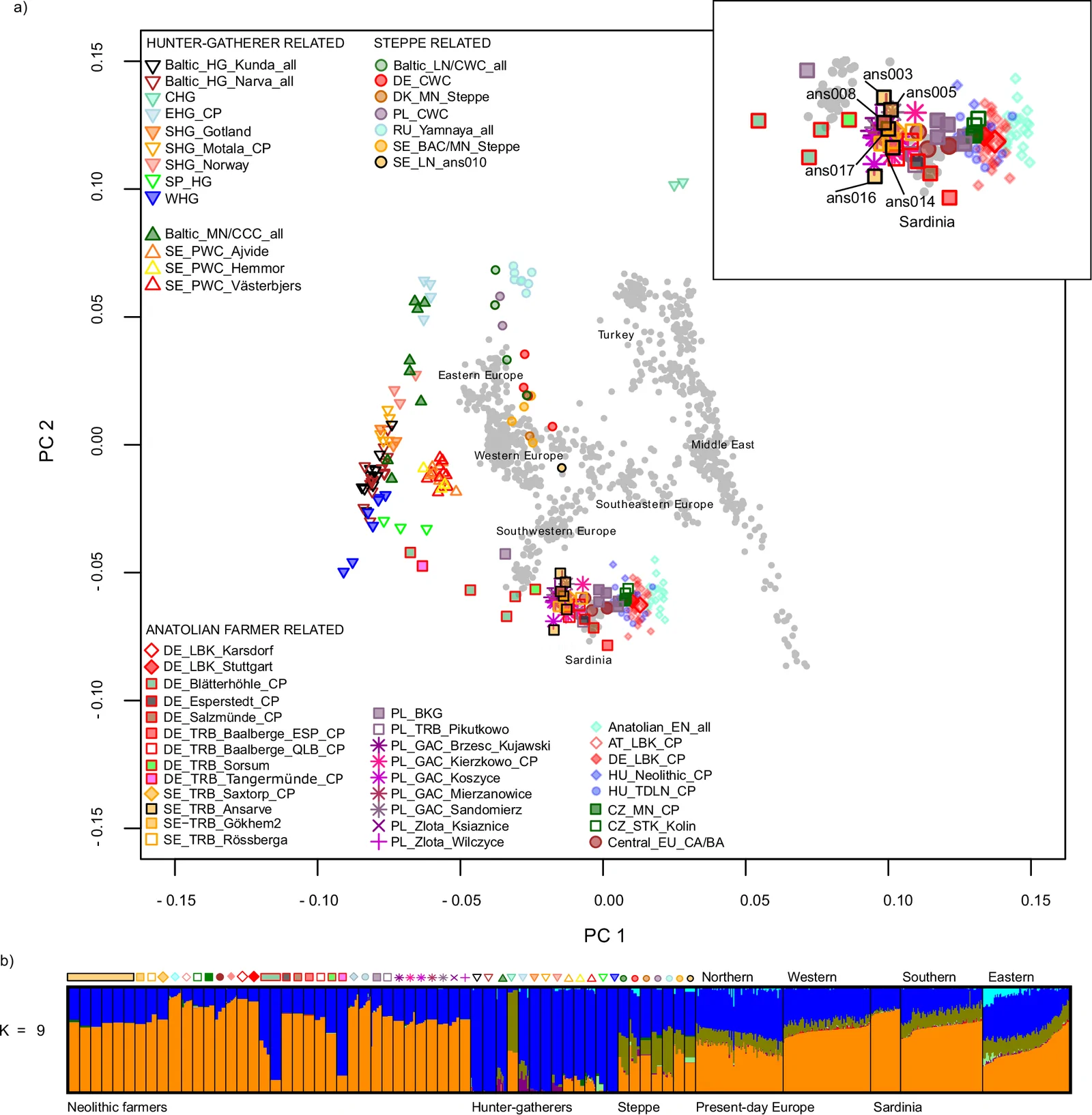
PCA and unsupervised admixture plot. **a** PCA of 991 West Eurasian present-day individuals (gray dots) from the Human Origins v2 data, where ancient 207 Hunter Gatherers, Neolithic Farmer, and Yamnaya/BAC/CWC are projected onto the first two principal components. A two-letter International Organization for Standardization format is used to represent the country of origin, plus abbreviated cultural name/ time-period(s), and site name (for groups)/ or lab names (for individuals). Most ancient samples are shotgun-generated; however, a suffix at the end of each label in the form of “_CP,” and “_all” depicts if cases where we used only capture-generated data or where both capture and shotgun data were merged. [see “Methods,” and Supplementary Section 4.1 for details]. **b** Unsupervised ADMIXTURE modeling results (*K* = 9) for ancient samples [Methods], and 923 individuals from 102 worldwide populations from the Human Origins v2 Panel [all Ks are shown in Supplementary Fig. 2]. The dark blue component is maximized in WHG, the orange component is maximized in Anatolian NFs, the green component is maximized in CHG, and Yamnaya. The plum and cyan component is related to Ancient North Eurasian ancestry, which is present in EHG and Steppe-related individuals. Labels correspond to [**a**].

We projected the Ansarve and the PWC individuals together with 207 published individuals with ancient genome-wide data, dated between the Mesolithic and Early Bronze Age [Supplementary Data 1], on top of the first two principal components using 991 present-day individuals from 67 European, Near Eastern, and Caucasian populations from the Human Origin v2 data^60,61^ [Methods]. Specifically, we compared the Gotlandic individuals to individuals from contemporaneous central European cultural groups and found that the Scandinavian TRB cluster together with individuals from the Globular amphorae (GAC) and Zlota cultural contexts in Poland in the PCA space [Fig. 2a], sharing similar levels of HG admixture [Fig. 2b and Supplementary Fig. 2]. Some contemporaneous NF individuals with elevated HG-admixture (e.g., Blätterhöhle, Tangermünde, and Sorsum) form a cline between the Scandinavian NF cluster and WHG. As previously shown^10^, the PWC individuals form a separate cluster separated from SHGs slightly in the direction of NFs caused by low levels of NF-admixture. Furthermore, the LN male individual from the Ansarve dolmen (SE_LN_ans010, 2030–1890 calBCE) falls in a cline together with other individuals that display Steppe-related ancestry, making him the earliest individual with Steppe-related ancestry found on Gotland to date.

**Fig. 3:**
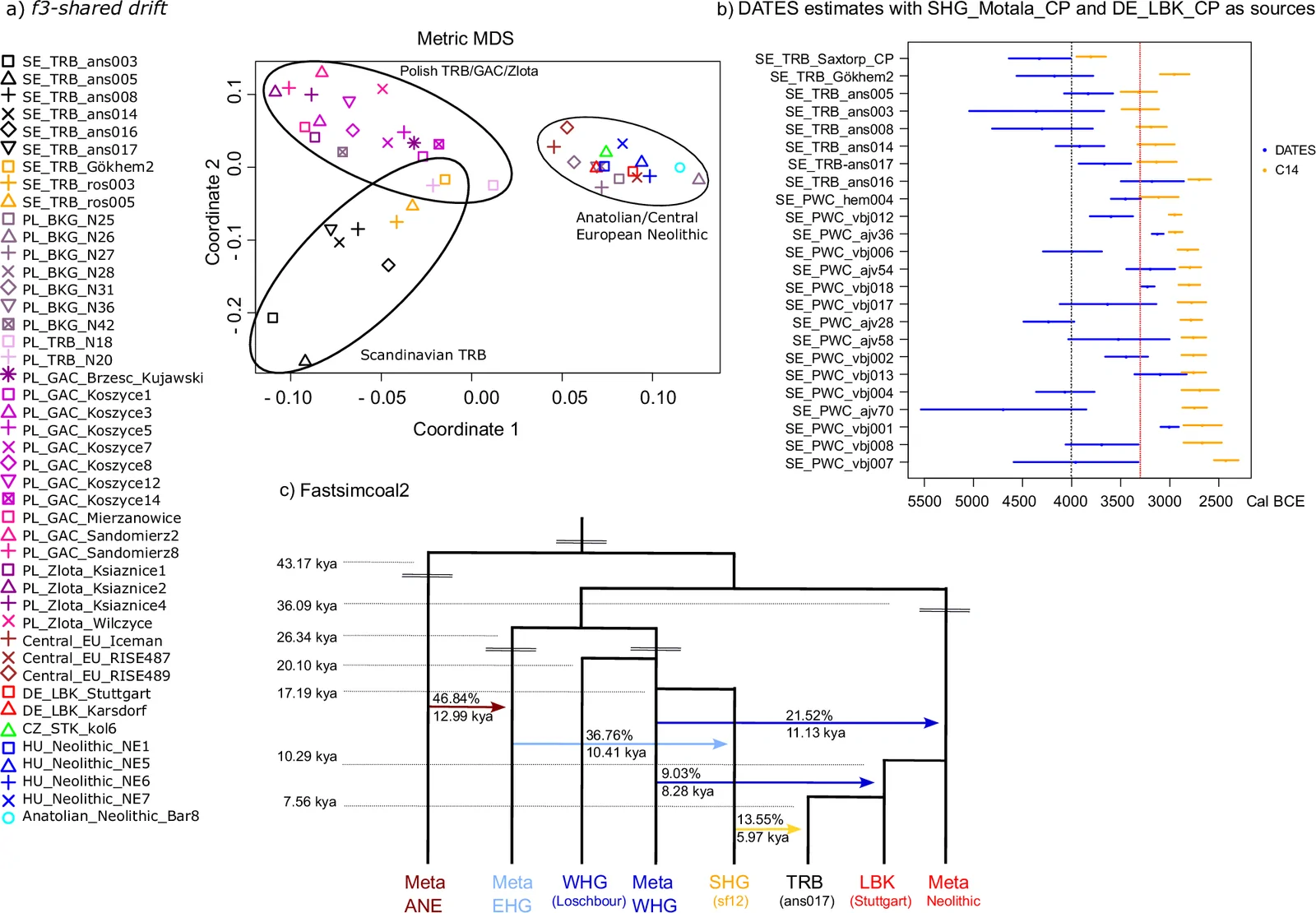
*f3*-shared drift MDS plot, DATES admixture estimation analysis, and demographic inference with *fastsimcoal2.* **a** MDS plot of *f3*-shared drift of the 1st and 2nd coordinates displays a connection between the Scandinavian and Polish Neolithic farmers to the exclusion of Central European Farmers [Supplementary Data 3]. The symbols in the figure are described to the left of the figure. The black ellipses depict farmers deriving from different geographical regions. **b** DATES analysis of timing of HG-, and NF-admixture in the Scandinavian TRB and PWC, respectively. Blue = point estimate admixture time in years (25 * generation) and SE, Yellow = point estimate for radiocarbon dates calBCE, 95.4% CI. Black dashed line = Neolithization of Scandinavia, Red dashed line = arrival on PWC material culture to Scandinavia and Gotland. **c** Demographic inference with *fastsimcoal2*—best-fitting model (# 4): WHG (Loschbour), SHG (sf12), ENF (Stuttgart), and SE_TRB_ans017 representing the Ansarve population. Unsampled meta populations labeled as “Meta ANE” (Ancestral North Eurasian), “Meta WHG,” “Meta EHG,” and “Meta Neolithic” with point estimates for branch split time, admixture event, and the admixture proportions [Modified from Supplementary Fig. 16C and Supplementary Data 10].

Further exploring the genetic structure among the NFs, we observed that the NF-individuals broadly separate into three main clusters of different geographic dispersal: (i) Scandinavian, (ii) Polish, and (iii) EN – MN Central European and Anatolian cluster [Fig. 3a], based on a shared-drift *f3-statistic*^61^ MDS analysis [Methods]. At the group level, we confirm the clustering of the Polish and Scandinavian TRB^62^; however, we also see a connection with the DE_TRB_Baalberge_QLB_CP individual [Supplementary Fig. 3]. At the individual level, we observe more genetic drift, indicating some degree of isolation among the Ansarve individuals, which differentiates them from Scandinavian mainland TRB contexts in Falbygden (Gökhem and Rössberga) [Fig. 3a]. The differentiation between the Ansarve individuals and TRB-associated individuals in the Falbygden area with > 250 megalithic burials can also partly be explained by the many familial relations found in that region^7^.

### Social structure and dynamics in Middle Neolithic Gotland

Previous studies demonstrated close kinship connections up to the 2nd degree in the Gotlandic TRB and PWC individuals^10,24^. Here, we also noticed that the two chronologically older females (ans003 and ans005) shared more drift with each other than with the other Ansarve individuals [*f4*-*statistics*, Supplementary Fig. 4]. We ran ancIBD^63^, which can detect kinship relations up to the 7th degree, and apart from the 2nd degree male relationship at Ansarve^24^, we found a 3rd degree relationship between the above-mentioned females [“Methods” and Supplementary Data 6]. However, there was not sufficient data to determine the nature of their relatedness, and the coverage for both individuals was below the recommended 0.4× cut-off value [Table 1].

We found kinship relations also in the three PWC groups and confirm the 2nd degree relatedness between SE_PWC_vbj001 and SE_PWC_vbj008^10^, here predicted to be grandparent-grandchild, and found another 3rd degree (great grandparent-grandchild) relation between SE_PWC_vbj006 and SE_PWC_vbj013. As well as several other kin relations from the 2nd to the 3–4th degree, both within and between all three sites, which suggest a long population continuity for PWC on the island and also contacts between the different sites [Supplementary Data 6].

We applied several different methods to understand the past population sizes of the different Stone Age groups [Methods]. From a PSMC analysis^64^ of the high-coverage genomes of SE_TRB_ans017, SHG_Gotland_sf12, WHG_Loschbour, and DE_LBK_Stuttgart, we found that the Ansarve TRB had a greater effective population size (N_e_) compared to SHGs and WHGs, but smaller compared to ENFs [Supplementary Fig. 5]. These observations are largely consistent with conditional nucleotide diversity estimation^5^, and also runs of homozygosity (ROH)^65^ analyses of imputed data (from >~1× genomes) [Supplementary Figs. 6–8], where the NF-individuals displayed broadly the same proportion of short (<1.6 Mb) ROH tracks along the genome with some slight variation on the amount of long (>1.6 Mb) ROHs.

The Mesolithic and Neolithic HGs, on the other hand, showed a higher proportion of short and long ROH tracks than the NF-groups [Supplementary Fig. 8], which implies that the HG-populations lived in smaller groups. The PWC individuals have a smaller proportion of these tracks than the Mesolithic HGs, probably as a consequence of the NF gene-flow. The lower N_e_ in the Gotland PWC individuals compared to the NFs was further confirmed from analyzing tracks of homozygosity (>4cM) and N_e_^66^ [Supplementary Fig. 9 and Supplementary Data 8 and 9].

However, the individual SE_TRB_ans017 showed a greater fraction of long ROH tracks (mostly above 12cM and longer) that also were present in most of the chromosomes, which is an indication of inbreeding, and he was also predicted to be the offspring of closely related parents^65,66^ [Supplementary Figs. 9–11]. This observation is in accordance with the inbreeding coefficient estimation (*F* = 0.10) for SE_TRB_ans017 [Methods], using both the high coverage genome and the 6.8× genome previously published^24^.

In order to assess the biological impact of the inbreeding in SE_TRB_ans017, we screened the high-coverage genome for deleterious variants [Methods]. Six variants were classified as “high impact” using SNPEff^67^ [Supplementary Table 6]; however, most of these variants either occurred at previously unknown variable sites, were present as heterozygotes, or their functional consequences are unknown [Supplementary Table 7], which complicates the interpretation of their biological implications.

### Admixture patterns analyses

As the Ansarve individuals display some level of gene-flow with HGs, and the PWC individuals display some level of gene-flow with NFs, and the two groups coexisted on Gotland for more than 500 years, we investigated if this admixture could have occurred during the period of coexistence on the island. It has previously been shown that NF admixed with WHGs during the continental expansion^4,5,68^. Thus, the ancestors of the Ansarve individuals exhibited evidence of WHG admixture prior to arrival to Scandinavia^8^. However, other studies have also proposed a later admixture between Scandinavian NF and SHG, e.g., ref. ^5^.

We used qpGraph^61^ [Methods] to model different scenarios. As we wanted to investigate HG admixture over time in the Ansarve TRB, we performed the analyses at the individual level for each Ansarve individual but investigated the PWC as one group. First, we tested a scenario with WHG, Anatolian_Neolithic, and SHG_Motala admixture into both Ansarve TRB individuals and into the PWC group, but without admixture between them [Supplementary Fig. 13]. Four out of the six Ansarve individuals (ans005, ans008, ans017, and ans016) are compatible with that model. Furthermore, these results also indicate that the Ansarve individuals exhibit between 9 and 21% gene-flow from a SHG-related population in addition to their Farmer/WHG admixed ancestry. Interestingly, this SHG-admixture seems to have increased with time when considering the radiocarbon dates of these individuals. The Ansarve individuals (ans003 and ans014) displayed less SHG-admixture (7 and 2%, respectively), but they were poorly fitted to the model.

Next, we assessed if the PWC group could be modeled as a mixture between SHG and the Ansarve TRB. This model would imply gene-flow between an Ansarve TRB-related source and the ancestors of the PWC (occurring either on Gotland or the mainland). Our results indicate that PWC individuals as a group can be modeled as having derived 31-36% Ansarve ancestry [Supplementary Fig. 14], likely acting as a proxy for farmer ancestry.

We then investigated closer genetic affinity [using a cross-coalescence rate approach^64^, “Methods”] between Ansarve_TRB (represented by the high-coverage genome ans017) and WHG_Loschbour, SHG_Gotland_SF12, plus DE_LBK_Stuttgart and found that SE_TRB_ans017 shared more recent ancestry with a SHG-like populations than with a WHG-like population [Supplementary Fig. 15].

Based on these results, we modeled the above-mentioned high-coverage genomes, together with unsampled ghost meta populations labeled as “Meta ANE” (Ancestral North Eurasian), “Meta WHG,” “Meta EHG,” and “Meta Neolithic using fastsimcoal 2.7”^69^ to infer the Ansarve demographic population history [Methods]. We tested four models: (1) No pulse admixture into the Ansarve population, (2) A WHG pulse admixture into the LBK+Ansarve ancestor, (3) A SHG pulse admixture into the Ansarve, and (4) A WHG pulse admixture into the LBK+Ansarve ancestor, plus a SHG pulse admixture into the Ansarve. We found that model (4) had the best likelihood [Fig. 3c, Supplementary Data 10 and Supplementary Fig. 16]. The total hunter-gatherer (SHG + WHG) ancestry in SE_TRB-ans017 amounts to 38% (95% CI: 11.24–44.71), consistent with the estimates inferred from qpGraph.

We then investigated the timing of HG-admixture into the Scandinavian TRB individuals [DATES^70,71^, “Methods” and Supplementary Data 11] and show that most of these gene-flow events with foragers occurred in their distant ancestors, either from a WHG-related population in the continent prior to the Neolithization of Scandinavia, and/or in Scandinavia with a SHG-related population prior to the appearance of the PWC [Fig. 3b]. Interestingly, SE_TRB_ans016, who displays the most SHG-ancestry (21%), also shows a more recent admixture event, after the appearance of the PWC-complex on Gotland. Furthermore, although the NF-admixture events seen in most of the PWC individuals happened in their distant ancestors, after the NF dispersal to Scandinavia and prior to the development of the PWC on Gotland, several individuals (SE_PWC_ajv36, SE_PWC_ajv54, SE_PWC_vbj001, SE_PWC_vbj013, and SE_PWC_vbj018) displayed more recent NF-admixture, which could have happened on the island.

### *Y. pestis* detection in the Ansarve and PWC samples

Lastly, we investigated the possibility of *Y. pestis* being present on Gotland at Ansarve and the PWC-sites^2,5,10,12,14,24,68^, and this study [“Methods” and Supplementary Section 7], given previous reports of *Y. pestis* in Scandinavia, the Baltic, and central Europe^7,55–58^.

Out of 783 libraries from 11 Ansarve and 21 PWC individuals, we found evidence for the possible presence of *Y. pestis* sequences in five individuals (SE_TRB_ans003, SE_TRB_ans005, SE_TRB_ans007^24^, SE_PWC_ajv58^14^, and SE_LN_ans010^12^ and this study, [Methods]. Since the number of mapping reads was too low to be able to perform robust phylogenetic analyses, we authenticated our results by combining different strategies; (i) competitive mapping against representatives of all known species of the *Yersinia* genus, (ii) edit distance profiles, (iii) number of reads mapping on chromosomes and plasmids, and (iv) the distribution of single-nucleotide variants (SNVs) among strains [Supplementary Tables 8 and 9 and Supplementary Fig. 17].

Our results supported, in all the analyses, the presence of *Y. pestis* in the LN individual (ans010; 2030–1890 calBCE), with a strain that most likely belonged to the LNBA-lineage defined in recent publications^58,72^. Membership to this lineage could be expected by the temporality of this sample. We also confirmed the finding of *Y. enterocolitica* previously detected in lower coverage data from this individual^73^, which could imply a coinfection with both species [Supplementary Data 13 and Supplementary Figs. 18 and 19]. *Y. enterocolitica* causes a possible lethal intestinal infection (yersiniosis) generally spread by infected water and food, commonly obtained from digesting raw or undercooked meat from suids^74^. A coinfection of the earlier Neolithic *Y. pestis strain* and *Y. enterocolitica* has previously been found in an MN individual from Falbygden^7^. Although we found evidence of *Y. enterocolitica* in several of the other individuals analyzed here, including the previously reported SE_PWC_ajv58^73^, there was too little data to confirm these results.

Similarly, authenticating the presence of *Y. pestis* was more challenging for the other four individuals. However, by combining the complementary evidence provided by the different authentication analyses we could verify the presence of *Y. pestis* in the early phase of the Ansarve burial (ans003; 3490–3110 calBCE), supported by the presence of reads mapping into the three typical plasmids of *Y. pestis* (pMT1, pPCP1 and pCD1), edit distance profiles closer to *Y. pestis* than to the closely related ancestor *Y. pseudotuberculosis*, and the presence of several SNVs that are typically characteristic of *Y. pestis* [Supplementary Table 8 and Supplementary Figs. 20 and 21]. Although the observed SNVs suggest an affinity to some of the most basal strains of *Y. pestis*, we could not determine which strain was the closest. We were not able to unambiguously validate the presence of *Y. pestis* in the other two MN individuals from the dolmen (ans005 and ans007); thus, higher sequencing efforts would be necessary to fully authenticate these results.

We did, however, find that *Y. pestis* was most likely present in SE_PWC_ajv58 (c. 2880–2630) from the Ajvide PWC site, as evidenced by several SNVs that are typically present in *Y. pestis* and not, for instance, in *Y. pseudotuberculosis* [Supplementary Fig. 21]. Moreover, we also identified several SNVs that suggest a closer affinity to the most basal RV2039 strain^55^ than to any other Neolithic and LNBA strain, suggesting that this individual was infected with a strain that is closer to the one infecting HGs in the Baltic.

## Discussion

Our analysis has generated unique insights into the lives and social networks of the descendants of the first Neolithic farmers (TRB) on the island of Gotland and their relationship to the contemporaneous marine HGs (of the PWC complex). These distinct archeological groups coexisted on the island for more than 500 years and had different demographic backgrounds, material culture expressions, subsistence economy, and lifestyles.

Regarding the genetic affinities among TRB groups, we confirm the connection between Ansarve and other TRB-associated individuals from Scandinavia and Poland^7,62^ but also find a connection with a TRB-associated individual from present-day Germany, suggesting a common genetic origin among groups of the TRB cultural sphere. The presence of the Ansarve dolmen confirms that the Gotland TRB group must have had contact with the larger TRB-complex, as they erected the monumental burial at the same time as they appear in Scandinavia and the continent, and more than 500 years after the initial appearance of TRB material culture on the island.

The Ansarve individuals seem to be genetically more similar to each other than to other TRB individuals and groups, likely due to the relative isolation on Gotland [Fig. 3a and Supplementary Fig. 4]. The presence of many TRB settlement sites and the abundance of TRB-associated stray finds across the island, do however suggest that there must have been a larger TRB population present, also confirmed by analyses of N_e_. Whether or not the Ansarve group is a good representative of this larger population remains unknown, since no other TRB-associated burial has been found on Gotland.

In addition to the previously shown male kin-relation (ans014 and ans017), we also find that the two females (ans003 and ans005) from the earliest use of the burial were likely related to the 3rd degree. These females could possibly be cousins, or great aunt/niece, although a strict matrilineal kinship relation can be excluded based on their mitochondrial haplotypes [Table 1]. Interestingly, even though the contemporaneous males (ans008 and ans014) share both Y chromosomal and mtDNA haplogroup lineages, they are not close kin related. However, as there were ~30 individuals in the burial, and at least 15 had previously been dated to the MN period^12^ there most likely were additional kin relations, as seen in the contemporaneous megalithic burials in mainland Sweden^7^. As previously shown^65,66^, SE_TRB_ans017 was predicted to be the offspring of 1st degree cousins, and showed signs of inbreeding (*F* = 0.10). Although rare, a few cases of inbreeding have also been found at other Neolithic farmer sites^7,65,75^. Thus, the kinship relations and close parental relatedness suggest that the Ansarve dolmen was the burial site of an extended family with long continuation over time.

We confirm previous reported kin-relations between PWC individuals^10^, as well as additional kin-relations, both within and between the three sites (Ajvide, Hemmor, and Västerbjers), suggesting close contacts between the different PWC groups in different regions of the island. We do observe a slightly higher *N*_e_ in the earlier individuals from Hemmor, compared to Ajvide and Västerbjers, which may be an indication of a bottleneck after the initial settlement, and subsequent isolation of the PWC population. This bottleneck does, however, not suggest elevated inbreeding in the later PWC individuals.

Contrary to the previous suggestion of no local gene-flow from SHG into the Scandinavian TRB^8^, we do find evidence of SHG-related gene-flow in the Ansarve individuals. They can be modeled with an additional 9–21% SHG admixture, in addition to their already NF/WHG admixed ancestry [Supplementary Fig. 13]. We find that these gene-flow events mainly happened prior to the appearance of the PWC material culture complex [Fig. 3b], with an SHG-related population either on the Swedish mainland, or possibly on Gotland, as there is some Late Mesolithic material culture evidence on the island^32^.

We found evidence of more recent HG gene-flow in the latest dated male Ansarve TRB-individual (ans016; 2810–2580 calBCE). As he displays non-local childhood Sr-signals^12^ this gene-flow event probably happened outside of Gotland. Nevertheless, recent HG admixture does not seem to have been common within the Scandinavian TRB, as only two females (FRA108 and ROS027) out of 105 investigated individuals from Falbygden in Västergötland were suggested to have recent HG-admixture^7^. Furthermore, these individuals, from three different megalithic tombs, date to the end of the TRB cultural phase, which, as previously suggested for Västergötland^7^, could indicate relaxed socio-cultural boundaries and/or a demographic decline, and that interactions between the TRB and PWC groups had become more common at this time.

Similarly, the majority of NF-admixture seen in the Gotlandic PWC individuals appears to have also happened after the initial dispersal of Neolithic groups to Scandinavia but prior to their appearance on Gotland. We do, however, find evidence of recent NF-admixture in several individuals from Ajvide and Västerbjers, which suggests ongoing gene-flow occurring between TRB and PWC groups. We show that the PWC (as a group), in addition to their SHG-ancestry, exhibit ~30% of NF/WHG admixed ancestry, which can be modeled as gene-flow from an Ansarve-related population. However, the exact admixture proportions are difficult to assess as the individuals from both groups were already admixed in different ways. We also did not detect any distant kin-relations between individuals from the two archeological groups.

The archeological record on Gotland shows that some long-range trade occurred, indicating contacts between the PWC and TRB. Though, there are no obvious indications of material culture hybridity of the two cultural complexes, as seen in the pottery styles at e.g., the Fagervik PWC site and at the Alvastra Pile dwelling on the Swedish mainland, e.g., ref. ^19^. Vanhanen et al.^33^ proposed that some mainland PWC-groups adopted cultivation from TRB farmers. However, on Gotland, it is rather in later dated PWC contexts, and after the TRB cultural phase had ended, that new agricultural elements such as grindstones and skeletal remains of domesticated animals are found^42,76^. Further analyses of additional PWC burials at the end of the TRB phase on Gotland are needed to clarify the level of NF gene-flow into the PWC.

Gotland’s placement in the center of the Baltic Sea and prehistoric maritime customs increased the chance of contracting disease from different geographical areas. Here, we were able to detect evidence of *Yersinia pestis* in three individuals from different time periods and different cultural affinities. The early TRB female (ans003; 3490 - 3110 calBCE) carried a lineage which, although not specified further, suggests affinity to a basal *Y. pestis* strain. As seen in Falbygden^7^, the Neolithic plague was widespread during this time.

We also report an infection by an LNBA-strain not previously observed in Scandinavia. The Late Neolithic male from the dolmen (ans010; 2030–1890 calBCE) was most likely infected with the LNBA-strain from the continent. He also had a coinfection of *Y. enterocolitica*^73^ and this study. Unfortunately, the cultural affinity of this individual is not known as he was interred in an older burial structure, but as he showed Steppe-related ancestry and a non-local childhood Sr-signal^12^ it could possibly be evidence of a plague transmission route. Though, the individual is represented by one tooth only, and due to the low genomic coverage of both the individual (0.06×) and the pestis genome, further analyses were not possible to conduct.

Interestingly, the third strain, found in a PWC male (ajv58; 2880-2630 calBCE) from Ajvide, suggests a closer affinity to the most basal RV2039 lineage found in Latvia^55^. This could indicate contacts to the east, which have previously been discussed based on similarities in PWC-artifacts and burial customs with the Late Combed Ware culture in the Baltic^19,77^. Interestingly, *Y. pestis* has also been detected in a contemporaneous dog from the same PWC site^57^. Although our results on *Y. pestis* and *Y. enterocolitica* are well-supported, further sequencing and capture-enrichment strategies will be needed to perform phylogenomic analyses that can provide a clearer picture on the strain diversity and their spread between these populations and regions.

In summary, we have examined the demographic history of two genetically and archeologically distinct Neolithic groups on Gotland (TRB farmers and PWC marine foragers) by analyzing their genetic variation. While the Gotland TRB share ancestry with contemporaneous TRB groups from present-day Scandinavia, Poland, and Germany, they also show some level of isolation. Furthermore, although we find some evidence of recent admixture between the Ansarve TRB and the Gotlandic PWC that could have happened on Gotland, we show that most of the interaction between the different groups appears to have happened in the past, and prior to PWC appearing on the island. We also find evidence of different *Y. Pestis* strains in individuals from both TRB and PWC on the island, as well as in a Late Neolithic individual buried in the Ansarve dolmen after the TRB phase. Thus, different factors could have aided in the decline of the TRB-complex on the island. However, the extent to which pathogens such as *Y. pestis* might have affected these populations on Gotland needs further investigation.

## Methods

### DNA extraction, library building, and sequencing

The ans017 and ans010 samples were prepared in facilities dedicated to analyses of ancient DNA [aDNA] at the DNA Laboratory DBW (De Badande Wännerna) at Campus Gotland, and at Human Evolution, Department of Organismal Biology (IOB), Uppsala University (UU), as previously presented^12,24^. Briefly, the surfaces of all bones and teeth were cleaned with bleach and irradiated with UV-light (6 J/cm^2^ at 254 nm) prior to sampling. A sample between 30 and 70 mg bone powder was drilled from a tooth root and digested overnight using the Yang silica-based extraction method^78^ modified using urea instead of sodium dodecyl sulfate^79^. One negative extraction control was included for every six samples. A total of three extractions were made for ans010 and thirteen extractions for ans017 [Supplementary Table 1 in Section 2]. Single-indexed Illumina multiplex DNA libraries were prepared using blunt-end (BE) ligation with P5 and P7 adapters, and amplification for 9–15 cycles using IS4 and index primers from Meyer and Kircher^80^. For this study, twelve additional Uracil-DNA glycosylase (UDG) treated damaged repaired Illumina Single indexed multiplex BE DNA libraries were produced on three of the previously generated extractions^12,24^ for ans017 following Meyer and Kircher^80^ and Briggs and Heyn^81^. All libraries were quantified on Bioanalyzer 2100 (High Sensitivity DNA chip) or 2200 TapeStation (High sensitivity DNA screentape), Agilent, following the manufacturer’s protocol and sequenced (or re-sequenced ans010) on Illumina Hiseq 2500 (v.4 chemistry and 125 bp paired-end reads) or HiSeq Xten (v2.5 chemistry and 150 bp paired-end reads) at the SNP & SEQ technology platform, UU. An additional eight double-indexed^82^ PCRs were generated for ans010 from three previously generated BE DNA libraries^12^ by the SciLife aDNA Unit, IOB, UU, following the same procedure as above and sequenced on the Novaseq X Plus system (XLEAP chemistry and 150 bp paired-end reads) [Supplementary Table 1 in Section 2.1].

### Data processing, authentication, and contamination control

Paired-end reads were merged using MergeReadsFastQ_cc.py^83^ if an overlap of at least 11 base pairs was found between forward and reverse reads, the base qualities were added together, and any remaining adapters were trimmed. Merged reads were then mapped single-ended with bwa aln 0.7.13^84^ to the human reference genome (build 37) using the following nondefault parameters: seeds disabled -l 16500 -n 0.01 -o 2^24^. To remove PCR duplicates, reads with identical start and end positions were collapsed using a modified version, to ensure random choice of bases, of FilterUniqSAMCons_cc.py^83^. Reads with less than 10% mismatches to the human reference genome, with a length longer than 35 base pairs, and with mapping quality higher than 30 were kept for downstream analyses. The Python script to process the data can be found at https://bioinf.eva.mpg.de/fastqProcessing/.

Authentication and contamination were estimated from read length [Supplementary Table S2] and deamination patterns [Supplementary Fig. 1], as well as from different contamination-estimation approximations applied to three data sources: [i] the mitochondrial genome^85,86^, [ii] the X chromosome if the individual was male^87,88^, and [iii] the autosomes^89,90^ [Supplementary Table 3].

### Biological sexing and uniparental markers

Sex determination for the Late Neolithic ans010 was conducted using the *Rx*^91^ method [Table 1]. The mtDNA haplotypes for SE_TRB_ans017 and SE_LN_ans010 were classified using Halplogrep 3 (v.3.2.1) and coincide with previously reported findings^12,24^ [Table 1]. The Y-chromosome haplogroups (Y-chr hg’s) for all male individuals in Table 1 were classified using all single base substitutions from the International Society of Genetic Genealogy (ISOGG; http://isogg.org) version 15.73, 11 July 2020. Published Y-chr hg’s classified from earlier ISSOGG versions were updated, and the Y-chr hg’s for SE_PWC_vbj013 and SE_PWC_vbj018^10^ have been restricted due to more stringent reporting in this study [see Supplementary Table S4 in Section 3.1 for more information].

### Panels and labels

Genetic data from the ancient individuals from the present study and previous publications [Supplementary Data 1] were overlapped with four different reference datasets by generating pseudo-haploid data. For each SNP site, a random read covering that site with minimum mapping and base quality of 30 was drawn (using Samtools 1.3 v mpileup), and its allele was assumed to be homozygous in the ancient individual (pseudo-haploid)^14^. Non-biallelic SNPs in the ancient individuals were excluded from the data. We only include ancient individuals with >0.05× genome coverage.

*Panel (1)*: we merged non-overlapping populations from Human Origins^60,61^ datasets comprising 616,938 SNPs genotyped in 2,582 modern individuals from 252 populations. Subsets of this dataset were used to perform PCA analyses and unsupervised ADMIXTURE v1.3^92^.

*Panel (2)*: 3,862,510 biallelic transversion SNPs with a minor allele frequency of at least 1% in worldwide populations from the 1000 genomes project^93^. This panel was used to perform *f-statistics* analyses using SG-generated data only.

*Panel (3)*: 1,938,919 biallelic transversion SNPs with a minor allele frequency of at least 10% in Yorubans (YRI) from the 1000 genomes project^93^ were used to perform CND analyses^14^, and also ROHs on imputed data^65,94^, and inbreeding coefficient^95^.

*Panel (4)*: autosomal SNPs from the 1.2 M SNP captured array^96^. Genetic data from all ancient individuals were screened for the 1,150,639 autosomal coordinates from this capture array, (and used for both SG- and CP-generated data). Ancient DNA data overlapped with this panel were used for qpGraph analyses^61^, DATES^70,71^, and HapRoh^66^.

The labels used for the ancient individuals in the demographic analyses are described in detail in Supplementary Section 4.1 and Supplementary Data 1.

### Principal component analysis

PCAs were performed using smartpca from the EIGENSOFT package, with the “numoutlieriter: 0” and “r2thresh: 0.2” parameters. For each ancient individual, a PCA was conducted together with 991 individuals from 67 European (EU), Near Eastern (NE), and Caucasian (Cau) populations extracted from the Human Origins panel v2^60,61^ and 207 published ancient genomes [Supplementary Data 1] dated between the Mesolithic and Early Bronze Age were plotted using Procrustes transformation, using all SNPs^2^. The result was plotted using an in-house R script from the vegan library. In the PCA [Fig. 2a], a two-letter code is used to represent the country of origin based on the two-letter International Organization for Standardization (ISO) for country format, plus abbreviated cultural name/ or the time-period(s), and site name (for groups)/ or lab names (for individuals). The Scandinavian samples represent whole-genome shotgun (SG) sequencing data only, comparative capture-generated data have an added “CP” tag, and the tag “all” is used when both are combined. See Supplementary Data 1 for more information on the cultures and nomenclature.

### Unsupervised ADMIXTURE

Ancestry components were inferred using ADMIXTURE v1.3^92^ based on 923 individuals representing 102 worldwide populations from the Human Origins v2 Panel^60^. Considering that our dataset harbors a large sample size of ancient individuals, we selected the 923 individuals to represent genetic variation across all continents. Supplementary Data 2 displays the present-day individuals used for this analysis. This subset of present-day individuals was merged with ancient individuals described in Supplementary Data 1. Only transversion SNPs were used in order to avoid biases due to cytosine deamination. The dataset was filtered for linkage disequilibrium using PLINK v1.90b4.988,89 with parameters (--indep-pairwise 200 25 0.4), which retained 75,789 SNPs. ADMIXTURE v1.3 was run in 20 replicates with different random seeds for ancestral clusters from *K* = 3 to *K* = 16. Common signals between independent runs for each *K* were identified using the LargeKGreedy algorithm of CLUMPP^97^. CLUMPP predicted that *K* = 9 was the highest K at which >80% of the runs were consistent in their ancestral component prediction. Clustering was visualized using Pong^98^ [Fig. 2b]. A description of the results across the different K’s can be found in Supplementary Section 4.2, and results for all K’s are shown in Supplementary Fig. 2 within the same section.

### *f-statistics*

To investigate the genetic structure among the ancient individuals from a farming context, we performed S*hared-drift f3-statistics* computation with the qp3Pop program in the ADMIXTOOLS package^61^ and using SGDP Mbuti individuals^93^ as an outgroup, using Panel 2 (for individuals) and Panel 4 (for groups) [Supplementary Data 3 and 4]. We then summarized information from each distance matrix on two dimensions using multidimensional scaling (MDS) with the “cmdscale” function in the R “stats” package (https://www.r-project.org) [Fig. 3a and Supplementary Fig. 3 in Section 4.3.1]. In both cases, individuals with low coverage per site were removed to avoid distortion of the MDS space. *f4-statistics* were computed with the same setup using only SG sequenced genome-wide data overlapped with Panel 2. We investigated a topology of the form *f4* (O, X; Anatolian Neolithic, SE_TRB_Ansarve individual) employing Mbuti as an outgroup, and X a Neolithic farmer [Supplementary Fig. 4 in Section 4.3.2 and Supplementary Data 5].

### ancIBD

We used ancIBD^63^ to infer kinship relatedness between 3rd and 7th degrees. Samples were imputed using GLIMPSE^99^ following author recommendations (https://odelaneau.github.io/GLIMPSE/docs/tutorials). Each sample was imputed separately to avoid batch effects. As a reference panel for imputation, we used the phased haplotypes from the 1000 Genomes dataset (http://ftp.1000genomes.ebi.ac.uk/vol1/ftp/release/20130502/). Imputed data and phased were used as input for ancIBD (v.0.2a; https:// pypi.org/project/ancIBD). As suggested by the authors, we down-sampled the imputed data to 1,240,000 SNPs and screened all pairs of Ansarve and PWC individuals with at least 0.4× coverage (*n* = 16) using ancIBD recommended default settings, to avoid spurious results between the lower coverage individuals. Results for each pair of individuals with IBD detected are displayed in Supplementary Data 6. However, we did include the lower coverage individuals SE_TRB_ans003 (0.14×) and SE_TRB_ans005 (0.13×) as they shared more drift with each other in the *f-statistics*.

### Effective population size per individual as a function of time “PSMC”

PSMC was inferred using the MSMC method^64^ v. 0.1.0. Analysis was performed on a dataset consisting of four present-day individuals from the HGDP (French, Han, Yoruba, and Karitiana)^100^, together with UDG-treated high-coverage data from this study (SE_TRB_ans017) plus four previously published individuals (SHG_Gotland_sf12, WHG_Loschbour, DE_LBK_Stuttgart [Supplementary Data 1], and Ust’_Ishim^101^. Input files were prepared using scripts provided with the release of MSMC, and MSMC was run with the nondefault parameters--fixedRecombination and -r 0.88 in order to set the ratio of recombination to mutation rate to a realistic level for humans. We plot the effective population size assuming a mutation rate of 1.25 × 10e-8 and a generation time of 30 years [Supplementary Fig. 5 in Section 5.1]. The curves for ancient individuals were shifted based on their average C14 date. Additionally, we used the multihetsep_boot-strap.py from the msmc-tools GitHub to generate 100 bootstraps per individual.

### Conditional Nucleotide Diversity

We investigated the conditional genetic diversity for each ancient population, estimating the average number of mismatches across all investigated sites with coverage between pairs of individuals^5,68^, only including ancient groups where we had data from at least two contemporaneous individuals per group. To avoid ascertainment bias, post-mortem damage, and to increase the number of sites, we used SNPs from Panel 3^68,93^, which only includes transversion variants. The standard error confidence interval was estimated from the distribution of mismatches for all pairs within a population using R [Supplementary Fig. 6 in Section 5.2].

### Imputation for analysis of runs of homozygosity (ROHs)

Imputation was carried out using Beagle 4.0^102^ for the ancient genomes of 55 individuals with depth coverage of >0.7×^103^ [Supplementary Data 7 and Supplementary Section 5]. Genotype likelihoods for biallelic autosomal SNPs in the 1000 Genomes phase 3 dataset (ftp.1000genomes.ebi.ac.uk/vol1/ftp/release/20130502) were called using the “UnifiedGenotyper” tool in “Genome Analysis Toolkit” (GATK) v3.5.0^104^. Non-biallelic sites with missing data, as well as genotypes probably derived from post-mortem damage, were excluded. Samples were merged by chromosome and imputed in 15,000 marker windows using the 1000 Genomes phase 3 haplotypic reference panel and genetic map files provided by the BEAGLE website (http://bochet.gcc.biostat.washington.edu/ beagle). To assess accuracy, imputed genotypes for down-sampled 1× genomes from high-coverage samples were compared to their diploid genotype calls [Supplementary Fig. 7 in Section 5.3].

### Runs of homozygosity (ROHs) using imputed data and high-coverage genomes

To further understand the demographic history of the Scandinavian farmers, in contrast to reference HGs and farmers, we estimated the ROHs from these individuals using the imputation data described above. ROHs were first investigated from imputed data in 55 ancient individuals following^65^. Imputed data were overlapped with a set of 1.9 M transversions from Panel 3 in order to maximize the information; monomorphic alleles had been previously removed using PLINKv1.90b4.9^105,106^ using the a –maf flag. The length and number of runs of homozygosity were estimated using PLINK v1.90b4.9, using the following parameters: --homozyg-density 50 --homozyg-gap 100 --homozyg-kb 500 --homozyg-snp 50 --homozyg-window-het 1 --homozyg-window-snp 50 --homozyg-window-threshold 0.05^94^. Individual ROH tracks were separated into two size bins, over and under 1.6 Mb. Long ROH (>1.6 Mb) are informative about recent patterns of inbreeding, while short ROH (<1.6 Mb) shed light on ancient population constrictions. Following recommendations from^65^ we estimated the fraction of the total genome under ROH for both track length categories and visually displayed the differences for each individual between each category of ROH [Supplementary Fig. 8 in Section 5.4.1].

### Estimating tracks of ROH from low-coverage data with hapROH

We investigated ROHs also in low coverage data (>0.3×) from using hapROH^66^ version 0.1a4 (https://pypi.org/project/hapROH), where we overlapped pseudo-haploid data on the 1240 K SNPs (Panel 4). We used default parameters and a default genetic map, and 5008 haplotypes from 1000 Genomes^93^ as a reference. We report the total sum of ROH >4, >8, >12, and>20 centiMorgan (cM) for the Scandinavian TRB and PWC [Supplementary Fig. 9 in Section 5.4.2]. Effective population sizes (Ne) were inferred both at the group and individual levels, including only individuals without inbreeding [Supplementary Data 8 and 9].

### Inbreeding coefficient for SE_TRB_ans017 high coverage data

Inbreeding coefficient was calculated following recommendations from Gazal et al.^95^ by estimating the fraction of the genome that is within homozygous-by-descent (HBD) in the high-coverage diploid data of SE_TRB_ans017, using ROH as a proxy for HBD^65^.

### Discovery and annotation of novel variants in the SE_TRB_ans017

We used GATK, v3.5.0, to discover novel variants in the high-coverage genome of SE_TRB_ans017. Indel realignment was performed using “RealignerTargetCreator” and “IndelRealigner” GATK’s modules. Prior to variant calling, the base quality of “T” nucleotides at the first five and “A” nucleotides at the last five positions of the sequencing reads was set to zero in their BAM file using a custom script from^14^ to prevent incorrect genotype calling due to residual deamination. “AddOrReplaceReadGroups” function of Picard (Broad Institute. Picard tools https://broadinstitute.github.io/picard/, 2016) was used to add read group. Indel realignment was performed using “RealignerTargetCreator” and “IndelRealigner” modules of GATK. Indels reported in the 1000 genomes project phase 1^107^ were used as a reference set for indel realignment in both “RealignerTargetCreator” and “IndelRealigner” modules with “--known” parameter. Genotype calling was conducted using GATK’s “UnifiedGenotyper” with expressions “-stand_call_conf 50.0 & -stand_emit_conf 50.0 & -mbq 30 & -contamination 0.02 & --output_mode EMIT_ALL_SITES.” dbSNP version 142 was used as known SNPs with the “--dbsnp” option. To filter the variants, the “VariantFiltration” module of GATK was used with expressions “--filterExpression ‘QD < 3.0 || FS > 60.0 || MQ < 35.0 || MQRankSum < −12.5 || ReadPosRankSum < −8.0 || MQ0 > = 5’ and --genotypeFilterExpression ‘DP < 10 || GQ < ${1} || DP > 120’.” Using BEDTools^108^, sites that are overlapping with 35 bp reads in Altai Neanderthal^100^ were extracted. Transition to transversion ratio was calculated using the “VariantEval” module of GATK with parameters [Supplementary Table 5 in Section 5.5]. Finally, variants with sequencing depth smaller than 25× were filtered using vcftools^109^. This retained a total of 1,672,348 variants. Of these, 6,267 are novel, and 1,667,081 are reported in dbSNP142. Functional annotation for all variants was performed using snpEff (v. 4.2)^67^. The proportion of both dbSNP-reported and novel variants in each functional annotation category is shown in Supplementary Fig. 12. Six of the novel variants were classified as “high impact” based on SnpEff [Supplementary Table 6]. Deleteriousness of the novel variants was evaluated using SIFT [Ng 2001] and Polyphen2^110^ [Supplementary Table 7].

### Admixture graphs qpGraph

To further characterize the demographic history of Ansarve and the Gotlandic PWC, as well as the proportion of gene-flow from different sources into these populations, we used qpGraph (version 6100)^61^ and overlapped genetic data from all relevant samples across all sites with Panel 4 and Mbuti as outgroup. The individuals used in the source groups are displayed in Supplementary Data 1. We accepted models with a |*Z*-score| < 3 values and excluded models with less than 15 000 SNPs (due to low power for rejection), or if models had inner zero-drift branches; however, final zero-drift branches were permitted [Supplementary Figs. 13 and 14 in Section 6.1].

### Divergence over time using the relative Cross Coalescence rate (rCCR)

To investigate the divergence over time between SE_TRB_ans017 and WHG_Loschbour, SHG_Gotland_SF12, and DE_LBK_Stuttgart, respectively, we used the relative Cross Coalescence rate (rCCR) and ran MSMC for two individuals (four haplotypes). We restricted this analysis to only UDG-treated genomes or down-scaled the first/last ten bases (when using non- UDG-treated libraries) to avoid potential biases from non-corrected deaminations. We considered a recombination rate of 0.88, a mutation rate of 1.25e-8, and a generation time of 30 years. To calculate the rCCR, we divided the cross-population coalescence rate by the mean of within-populations coalescence rate, which displayed how close the populations were at a given time point [Supplementary Fig. 15 in Section 6.2].

### Fastsimcoal modeling

Demographic inference of the Ansarve population’s history was conducted using fastsimcoal2.7^69^ in accordance with the methods outlined by Marchi et al.^111^. This analysis was performed on a panel of high-coverage genomes of ancient western Eurasian individuals, with WHG_Loschbour representing WHG, SHG_Gotland_sf12 representing SHG, DE_LBK_Stuttgart representing ENF, and SE_TRB_ans017 representing the Ansarve population. The dataset was filtered to exclude any sites with missing data, sites where the reference alleles differ between chimpanzee and gorilla reference genomes, CpG sites or regions, and sites located in genomic regions with a recombination rate below 1 cM/Mb. To focus on neutral sites suitable for demographic inference, only BGC-free A>T and G>C polymorphic sites were retained, resulting in a final panel consisting of 112,366,543 sites.

Four models were tested: (1) no pulse admixture into the Ansarve population, (2) WHG pulse admixture into LBK + the ancestors of Ansarve, (3) SHG pulse admixture into Ansarve, and (4) WHG pulse admixture into LBK+ the ancestors of Ansarve, plus SHG pulse admixture into Ansarve [Supplementary Data 10 and Supplementary Fig. 16A in Section 6.3]. Unsampled meta populations labeled as “Meta ANE” (Ancestral North Eurasian), “Meta WHG,” “Meta EHG,” and “Meta Neolithic” were also incorporated into the models. For each model, parameter estimates were obtained by maximizing the model’s likelihood across 50 independent runs of fastsimcoal, each involving 50 Expectation Conditional Maximization (ECM) cycles and 500,000 coalescent simulations to estimate the expected Site Frequency Spectrum (SFS) (command line used for this step was: fsc -t Panel.tpl -n500000 -d -e Panel.est -M -L50 -q -C5 --multiSFS --logprecision 18 -c16 -B16).

For each model, the maximum likelihood (ML) parameters were recorded based on the run with the highest likelihood from the 50 independent runs [Supplementary Fig. 16B]. The relative likelihood and Akaike Information Criterion (AIC) were calculated for model comparison. We further validated the results by comparing likelihood distributions using 10 million coalescent simulations, repeated 100 times (command line used for validation: fsc -i Panel_maxL.par -R100 -n10000000 -d -u -C5 --logprecision 18 -q -c16 -B16).

To obtain confidence intervals around the ML parameter estimates for the best-fitting model (WHG and SHG pulse admixture into Ansarve) [Fig. 3c and Supplementary Fig. 16C], a parametric bootstrap approach was employed. Using the estimated ML parameters, we generated 100 SFS (command line used was: fsc -i Panel.par -n100 -j -d -s0 -x –I -q -u -c16 -B16). For each of the 100 bootstrapped SFS, parameters were re-estimated with 20 independent runs, starting from the ML parameter values, and each run consisted of 60 ECM cycles with 500,000 simulations to estimate the SFS and model likelihood (command line used was: fsc -t Panel.tpl -n500000 -d -e Panel.est --nitvalues Panel.pv -M -L60 -q -C5 --multiSFS --logprecision 18 -c16 -B16). The 95% confidence intervals were determined by calculating the 2.5% and 97.5% quantiles of the parameter distribution across the 100 re-estimated ML parameters.

We estimated the total HG ancestry (SHG + WHG) in SE_TRB_ans017 based on the model with the highest likelihood [Supplementary Data 10]. Initially, the Meta Neolithic population received an influx of WHG ancestry at 21.52% [95% CI: 3.44–25.98%]. Later, the common ancestor of Ansarve and LBK experienced an additional WHG ancestry influx of 9.03% [95% CI: 3.73–13.16%]. This resulted in a total WHG ancestry of **28.61%** [95% CI: 7.04–35.72%], calculated as: 9.03 + (100 − 9.03) × 0.2152 = 28.61%. Furthermore, Ansarve received an additional SHG admixture of 13.55% [95% CI: 4.52–13.99%]. This led to a total hunter-gatherer ancestry of **38.27%** [95% CI: 11.24–44.71%], calculated as: 13.55 + (100 − 13.55) × 0.286 = 38.27%.

### Admixture DATES estimate

To investigate the timing of the Admixture in the Scandinavian TRB and PWC, we used DATES^70,71^ and genetic data overlapped with all sites from Panel 4. We used default parameters as suggested in the GitHub default web page (https://github.com/priyamoorjani/DATES). SHG_Motala_CP and DE_LBK_CP were used as proxy populations [Supplementary Data 11] as we wanted to have data generated from the same methodology with a large sample size that would allow us to measure the time to admixture for both Scandinavian farmers and PWC simultaneously. We assumed 25 years per generation, and disregarded results when a negative number of generations was observed or when the estimated standard error was superior to the estimated number of generations. [Fig. 3b]. We also investigated to date the time of admixture using WHG_CP and DE_LBK_CP as proxy populations; however, the results generally suggested much older dates [Supplementary Data 12].

### Metagenomic analysis

We screened 180 aDNA libraries from 11 individuals from the Ansarve burial and 603 libraries from 21 PWC individuals, in order to assess if we could identify pathogens in the metagenomic data for Gotland’s farmer and forager communities [Supplementary Section 7]. We used MALT^112^ to screen the metagenomic data, using a reference genome collection that contains archaeal, bacterial, fungal, and viral species (*n* = 6133) from reference and representative genomes of the RefSeq database^113^. Before indexing the reference genomic collection, Dustmasker^114^ was used to mask repetitive regions of the full-length reference genomes to avoid false positive classifications. Then we aligned DNA reads to the indexed reference genome collection using MALT with the semi-global flag on. This tool aligns DNA reads to the reference genome collection and, using a “lowest common ancestor” method, assigns sequences to a specific taxonomical node. We used MEGAN^115^ tool to process MALT’s output to obtain species absolute abundance results. Our initial analysis showed *Y. pestis* signals in ans003, ans005, ans007, ans010, and ajv58 [Supplementary Table 8]. We further mapped metagenomic reads from putative pathogens to *Y. pestis* (strain NW56), *Y. enterocolitica* (strain CO92) and *Y. pseudotuberculosis* (strain IP32953) reference genomes using bwa, forcing reads to map on their entire length (-l 1024), with a mapping quality >30 (-q 30) and eliminating reads shorter than 30 bp; duplicate reads were then removed using picard tools *MarkDuplicates*, as described by Key et al.^116^ [Supplementary Table 9, Supplementary Figs. 17–20, and Supplementary Data 13]. Given that MALT results suggest the presence of Y. pestis, this study only investigated the presence of this pathogen, as well as closely related strains to it in this study. A heatmap plot of shared derived SNVs between mapped reads from the samples of this study and 79 ancient and modern *Y.pestis* sequence was generated with R using package heatmap2^117^ [Supplementary Fig. 21].

### Statistics and reproducibility

Demographic statistical analyzes such as *f3*, *f4-statistics* and *qpGraph* were performed downloading publicly available scripts/software for Admixtools v1 (https://github.com/DreichLab/AdmixTools), smartpca from the EIGENSOFT v8 package (https://github.com/DreichLab/EIG), ADMIXTURE v1.3 (https://dalexander.github.io/admixture/), ancIBD (https://github.com/hringbauer/ancIBD) for kinship analyses, hapROH v1 (https://github.com/hringbauer/hapROH) for Runs of Homozygosity (ROHs) analyses in low-coverage genomes, DATES v1 software (https://github.com/priyamoorjani/DATES) for time-to-admixture dating estimates, the effective population size over time estimations and relative Cross Coalescence rate using the MSMC software (https://github.com/stschiff/msmc-tools), and Ansarve’s population history modeling inference using fastsimcoal v2.7 (https://speciationgenomics.github.io/fastsimcoal2/). We generated genotype likelihood estimations, as well as genotype calls using GATK, v3.5.0 (https://gatk.broadinstitute.org/hc/en-us), and ROHs analyses using imputed data and high-coverage genomes using PLINKv1.90b4.9 (https://www.cog-genomics.org/plink/). MALT, Dustmasker, Picard tools, and MEGAN were used for metagenomic analyses. No statistical analyses using other software were performed.

## Supplementary information

Supplementary material of the manuscript

Description of Additional Supplementary Files

nr-reporting-summary PDF

Supplementary Data 1–13

## Data Availability

Newly generated human and bacterial genomic sequences have been deposited in the European Nucleotide Archive under the accession PRJEB112372. The human reference genome (hg19) used during alignment is available via the 1000 Genomes Project^93^ repository (https://ftp.1000genomes.ebi.ac.uk/vol1/ftp/technical/reference/phase2_reference_assembly_sequence/).
