# Supplementary material for: Ancestry, admixture, and pathogens in contemporaneous Neolithic farmers and foragers on the Island of Gotland
Source: Commun Biol. 2026 Jul 13;9:972. doi: 10.1038/s42003-026-10498-0 (PMC13376394; doi:10.1038/s42003-026-10498-0)
Supplement: Supplementary file 2 — Description of Additional Supplementary Files [file 42003_2026_10498_MOESM2_ESM.pdf]

Description of Additional Supplementary Files

File name- Supplementary Data 1-13

Description - This excel file has the Supplementary Data tables, ordered from 1 to 13. We incorporate this "extended" tables in a separate excel file since they were too big to fit in a word file page. We preferred that all information relevant to a given section is displayed together.
